# Clinical application of the HM-1000 image processing for HER2 fluorescence in situ hybridization signal quantification in breast cancer

**DOI:** 10.1186/s13000-024-01455-8

**Published:** 2024-02-15

**Authors:** Vicente Peg, Teresa Moline, Miquel Roig, Yuko Saruta, Santiago Ramon y Cajal

**Affiliations:** 1 Pathology Department, Vall d’Hebron University Hospital, Passeo Vall d’Hebron, 119-129, 08035 Barcelona, Spain; 2 Autonomous University of Barcelona, Barcelona, Spain; 3 Spanish Biomedical Research Centre in Cancer (CIBERONC), Madrid, Spain; 4 Sysmex R&D Center Europe GmbH, Hamburg, Germany

**Keywords:** Breast cancer, Fluorescence in situ hybridization, *HER2* expression, Super-resolution microscopy

## Abstract

**Background:**

Accurate quantification of human epidermal growth factor receptor 2 (*HER2*) gene amplification is important for predicting treatment response and prognosis in patients with breast cancer. Fluorescence *in situ* hybridization (FISH) is the gold standard for the diagnosis of HER2 status, particularly in cases with equivocal status on immunohistochemistry (IHC) staining, but has some limitations of non-classical amplifications and such cases are diagnosed basing on additional IHC and FISH. This study investigated the clinical utility of a novel super-resolution fluorescence microscopy technique for the better FISH signal visualization and HER2 FISH classification.

**Methods:**

Fourteen breast cancer tissue samples were retrospectively collected between September 2018 and February 2022, and FISH *HER2* signal quantification was evaluated by determining the *HER2*/chromosome 17 centromere (CEP17) ratio and the number of *HER2* signals per nucleus in super- versus conventional-resolution images.

**Results:**

Super-resolution images maintained the same overall *HER2* diagnosis from routine, but *HER2* FISH amplification changed negative to monosomy in two cases. Two Letrozole non-response relapses coincided to monosomy samples. The median number of *HER2* signals per nucleus was 7.5 in super-resolution images and 4.0 in conventional-resolution images in HER2-positive samples and 2.8 and 2.1 signals per nucleus, respectively, in HER2-negative samples.

**Conclusions:**

Super-resolution images improved signal visualization, including a significant difference in the number of countable *HER2* and CEP17 signals in a single nucleus compared with conventional-resolution images. Increased accuracy of signal quantification by super-resolution microscopy may provide clinicians with more detailed information regarding HER2 FISH status that allows to better FISH classification such as *HER2*-low samples.

## Background

In patients with breast cancer, evaluation of human epidermal growth factor receptor 2 (HER2) expression is essential to determine whether targeted anti-*HER2* treatment is appropriate. In routine clinical practice, HER2 expression status is tested by immunohistochemistry (IHC) and fluorescence *in situ* hybridization (FISH). Currently, FISH is more commonly used for evaluation of HER2 status and is now included as part of the standardized criteria for diagnosis in the 2018 American Society of Clinical Oncology (ASCO)/College of American Pathologists (CAP) HER2 testing guideline update [1]. Interpretation of FISH is based on quantification of *HER2* and chromosome 17 centromere (CEP17) gene amplification signals [2]. Several studies have provided evidence of the correlation between *HER2* amplification by FISH and response to anti-HER2 treatment [3–5]. These studies demonstrated that correct quantification of *HER2* amplification, especially in samples with high HER2 expression, is important for predicting treatment response and patient prognosis in both the adjuvant and neoadjuvant setting.

Diagnosis of HER2 status based on FISH is the gold standard for cases with equivocal status on IHC staining, but there are some limitations associated with evaluation and signal quantification due to the low image quality and resolution [6]. When using a conventional fluorescence microscope, signals are sometimes blurred or overlapped with other signals. This can happen when the *HER2* signals show an amplification cluster, the samples have high background noise and weak signals, or the signals at different depths are blurred or not visible at the same focus level. Ambiguity of *HER2* signal images can cause inter-observer variability and uncountable or inaccurate results. In the past two decades, automated quantification of FISH signal amplification for HER2 status has been developed as an approach to solve some of these problems [7–10]. Different image analysis software and deep-learning systems have been established, but the fundamental limitation related to resolution and background noise due to autofluorescence in sample tissues remains unresolved.

Super-resolution microscopy is a series of technologies currently used in basic and advanced research, with some technologies achieving approximately 20-nm resolution [11]. Nanoreso (Sysmex, Kobe, Japan) is one such technology that captures the autonomous blinking of fluorophores in thousands of image frames and creates super-resolution fluorescent images based on Gaussian fitting of bright fluorescent spots. Nanoreso super-resolution microscopy normalizes the FISH signal without the need for additional staining and saves the captured images as a compatible digital file. This technology improves image resolution and provides a digitalized image for standardized quantification and signal localization that may be applicable for use in daily clinical practice.

## Material and methods

The aim of this study was to compare *HER2* signal quantification between conventional-resolution and super-resolution images using the HM-1000 (Sysmex, Kobe, Japan), a single-molecule fluorescence microscope with applied Nanoreso technology. The HM-1000 microscope may afford more precise quantification of *HER2* amplification in patients with breast cancer, thereby providing better categorization of HER2 status for therapeutic decision making.

Retrospective breast cancer tissue samples with FISH results from routine practice were collected between September 2018 to February 2022 at the Anatomic Pathology Laboratory in Hospital Vall d’Hebron, Spain. Patient data were collected and anonymized before the analysis was performed. The samples were previously tested by IHC staining and FISH based on the 2018 ASCO/CAP HER2 testing guideline update [1]. *HER2* FISH status was achieved by evaluating the *HER2*/CEP17 ratio and the number of *HER2* signals per nucleus with super-resolution and conventional-resolution images.

### FISH imaging

The collected tissue samples were de-paraffined and stained by dual-probe FISH for *HER2* and CEP17 (Cytocell Ltd, Cambridge, UK) which the concentration of *HER2* probe was 13.5 ng/μL and the CEP17 probe was 4 ng/μL, and by 0.125 µg/mL 4',6-diamidino-2-phenylindole (*DAPI*) for cell nuclei in 3-μm thick samples according to the following protocol.

Pre-treatment of samples was conducted using the Cytocell Tissue Pretreatment kit (RLPS100), in which the pre-treatment solution was applied for 30 minutes at 98°C and the samples underwent enzyme digestion with pepsin at 37°C for 20 minutes. Next, 15 μL of Cytocell *HER2* (ERBB2) Amplification (LPS 001) probe was applied, and the samples were denatured at 75°C for 5 minutes and hybridized at 37°C overnight. After hybridization, the slides were washed with astringent solution 0.4× saline sodium citrate (SSC) solution (pH7.0) at 72°C for 2 minutes and 2× SSC + 0.005% Tween-20 solution (pH7.0) for 30 seconds.

Before FISH imaging, 15 μL of *DAPI* diluted in 1:5 by the imaging buffer for Oxygen scavenging were added to the samples, which were then each sealed with a cover glass.

### Image digitalization

*HER2* and CEP17 signals were captured using the HM-1000 fluorescence microscope with 100x/1.4 oil-immersion objective lens. Using 40% laser intensity, the excitation wave of each gene marker fluorochrome was 488 nm and 561 nm, respectively. The exposure time was 30 ms per frame, and 5000 frames per field were captured to reconstruct the super-resolution image to approximately 20-nm resolution. For nuclei imaging, the 405-nm excitation wave with 20% laser intensity was used and 1000 frames per field were captured. To select the optimal sample field, we utilized the 10 × 10 tiling mode of the HM-1000 software to obtain the image data for 100 fields. The captured images were automatically saved as TIFF files.

### Digital image processing

The captured images were processed in four steps by machine learning and prediction techniques to reduce autofluorescence. First, the image file, which contained 5000 frames captured by the HM-1000, was separated into single image files using Python programming language. A total of 15,000 image files from three samples were used for Noise2Void model training, with the training images stored in TensorFlow [12, 13]. Sample images for the study were also separated in single image files and applied to the trained model to optimize the noise on each file using ImageJ software (U.S. National Institutes of Health, Bethesda, USA). Second, background noise was estimated using the rolling-ball algorithm plug-in and subtracted to obtain cleaned images (Castle and Keller [14]). Third, background thresholds were determined based on a linear regression model and machine learning. Finally, the separated 5000 image files were combined into one file using Python and adjusted by the slice-keeper function of ImageJ. Using ThunderSTORM, the modular plug-in for ImageJ that is used for sub-diffraction localization of molecules [15], Gaussian normalization was applied to each image, which consisted of 5000 noise-reduced frames, to create the super-resolution image. All developed scripts are available in the website GitHub [16].

### Signal quantification

Fluorescence signals were counted manually by two observers. The nuclei with clustered or ambiguous signals were estimated from the distinguishable signal count or defined as more than four signals. *HER2*/CEP17 ratio was calculated from the number of nuclei in which CEP17 signals were countable. HER2-positive or -negative status for the images using conventional-resolution and super-resolution were based on the 2018 ASCO/CAP HER2 testing guideline update [1]. Conventional-resolution was defined as the image captured using the HM-1000 fluorescence microscope without digital image processing.

### Statistical analysis

Commercialized software for statistical analysis, NCSS 10 (NCSS LLC., Kaysville, Utah, USA) was used for the analyses. Descriptive statistics, including mean, standard deviation (SD), median and range for continuous variables and frequency and percentage for categorical variables, were used to present the data. Normality tests were performed to define the statistical method for each significance test. Statistical significance (*p-*value) between two independent subgroups was determined using non-parametric tests: the Mann–Whitney U test was used for ordinal or continuous independent variables, the Chi-squared test for nominal independent variables, and the Wilcoxon test for paired samples. A *p-*value of <0.05 was considered statistically significant. The effect of each parameter was presented using a dot plot, percentage and 95% confidence intervals.

## Results

### Tissue samples

A total of 14 formalin-fixed, paraffin-embedded tissue samples were analyzed. The main characteristics of the patients in this study are summarized in Table 1. According to the routine hospital FISH-based diagnosis, nine samples (64.3%) were HER2 negative and five (35.7%) were HER2 positive. The overall *HER2* diagnosis integrating IHC and FISH showed no difference between conventional-resolution image and super-resolution image using HM-1000–captured images. Demographic variables showed no significant differences between HER2-positive and HER2-negative patients. In HER2-positive samples, the median *HER2* signals per nucleus was 7.5 in super-resolution images and 4.0 in conventional-resolution images. In HER2-negative samples, there was a median of 2.8 *HER2* signals per nucleus in super-resolution images and 2.1 signals per nucleus in conventional-resolution images.

**Table 1:** Patient characteristics by HER2 status according to routine diagnosis

	**Total**	**HER2-positive**	**HER2-negative**	***p*-value**
Age, years				
Median (min; max)	75.5 (51.0; 87.0)	67.0 (54.0; 86.0)	82.0 (51.0; 87.0)	0.46^a^
Q1; Q3	55.5; 84.25	55.0; 79.5	61.5; 84.5	
pT, n (%)				
pT1	5 (35.7)	2 (14.3)	3 (21.4)	1.00^a^
pT2	7 (50.0)	2 (14.3)	5 (35.7)	
pT3	1 (7.1)	1 (7.1)	0	
pT4	1 (7.1)	0	1 (7.1)	
Tumor size, mm				
Median (min; max)	22.5 (10.0; 70.0)	23.0 (14.0; 70.0)	22.0 (10.0; 31.0)	0.35^a^
Q1; Q3	17.0; 30.3	18.0; 52.5	15.5; 28.5	
Tumor type, n (%)				
NST	11 (78.6)	5 (35.7)	6 (41.9)	0.15^b^
ILC	3 (21.4)	0	3 (21.4)	
pN, n (%)				
0	8 (57.1)	3 (21.4)	5 (35.7)	0.87^b^
1	6 (42.9)	2 (14.3)	4 (28.6)	
Systemic therapy, n (%)				
Response	11 (78.6)	4 (28.6)	7 (50)	0.92^b^
Non-response	3 (21.4)	1 (7.1)	2 (14.3)	
Follow-up status, n (%)				
Relapse	2 (14.3)	0	2 (14.3)	0.23^b^
Death	2 (14.3)	1 (7.1)	1 (7.1)	
HER2 status (IHC), n (%)				
1+	7 (50.0)	0	7 (50.0)	<0.01^b^
2+	3 (21.4)	1 (7.1)	2 (14.3)	
3+	4 (28.6)	4 (28.6)	0	
HER2 status (SR^c^), n (%)				
Positive	5 (35.7)	5 (35.7)	0	<0.001^b^
Negative	9 (64.3)	0	9 (64.3)	

*FISH* fluorescence *in situ* hybridization, *HER2* human epidermal growth factor receptor 2, *IHC* immunohistochemistry, *ILC* invasive lobular carcinoma, *max* maximum, *min* minimum, *NST* invasive carcinoma of no special type, *Q1* first quartile, *Q3* third quartile, *SR* super-resolution

Clinicopathological characteristics were compared according to HER2 status determined by routine diagnosis; IHC and conventional-resolution FISH images according to the 2018 ASCO/CAP HER2 testing guideline update

^a^Mann–Whitney U test

^b^Chi-squared test

^c^HER2 status determined by super-resolution FISH images according to the 2018 ASCO/CAP HER2 testing guideline update

### Noise optimization

**Fig. 1:**
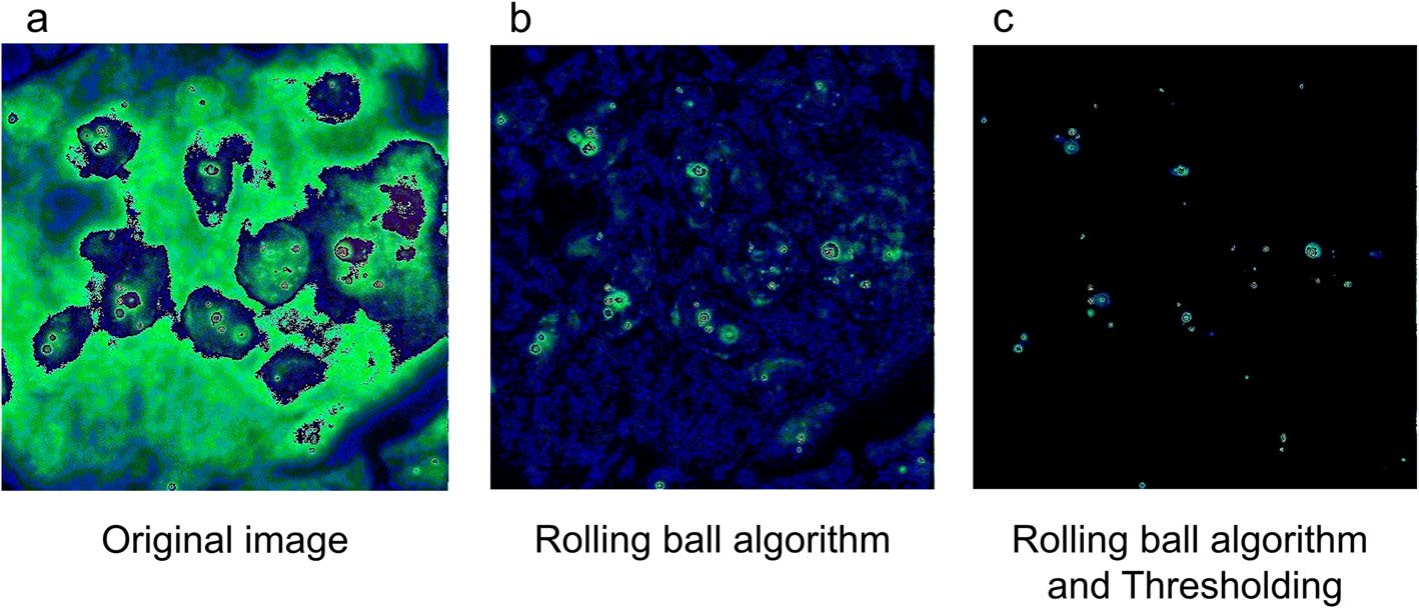
Noise optimization by machine learning model. Noise optimization and background subtraction for CEP17 signals, showing (**a**) the original image colored in 3-3-2 RGB; (**b**) the image after application of the rolling ball algorithm, where the radius pixels used for background subtraction is 15; and (**c**) after application of the threshold (1490), which was automatically obtained from the linear regression model

**Fig. 2:**
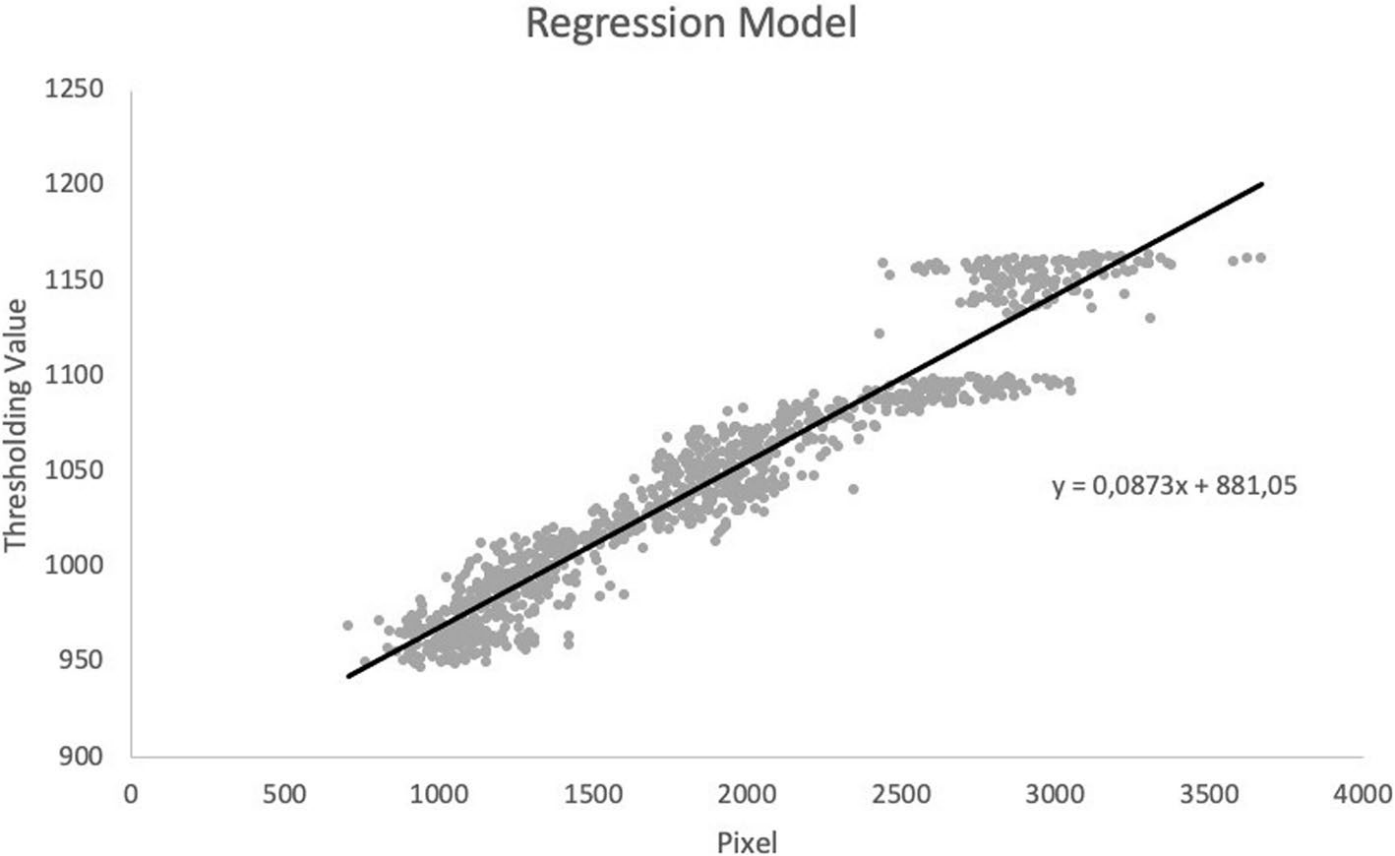
Linear regression model used to estimate the optimum background threshold. The preset linear regression model was used to determine the optimum threshold, then the reverse threshold was determined and the >95% correlation between the optimum threshold value and the mean (standard deviation) of the image was calculated. The optimum threshold value of each image was estimated using this regression model

As ThunderSTORM is very sensitive to background noise, noise reduction was applied for correct sample observation. The noise reduction process was performed using a machine learning model (Fig. 1). The original image included high background noise, which obstructed visualization of the *HER2* signal. After application of the rolling-ball algorithm and the background threshold, significant noise reduction was observed. The background threshold for each image was estimated using the linear regression model (Fig. 2).

**Fig. 3:**
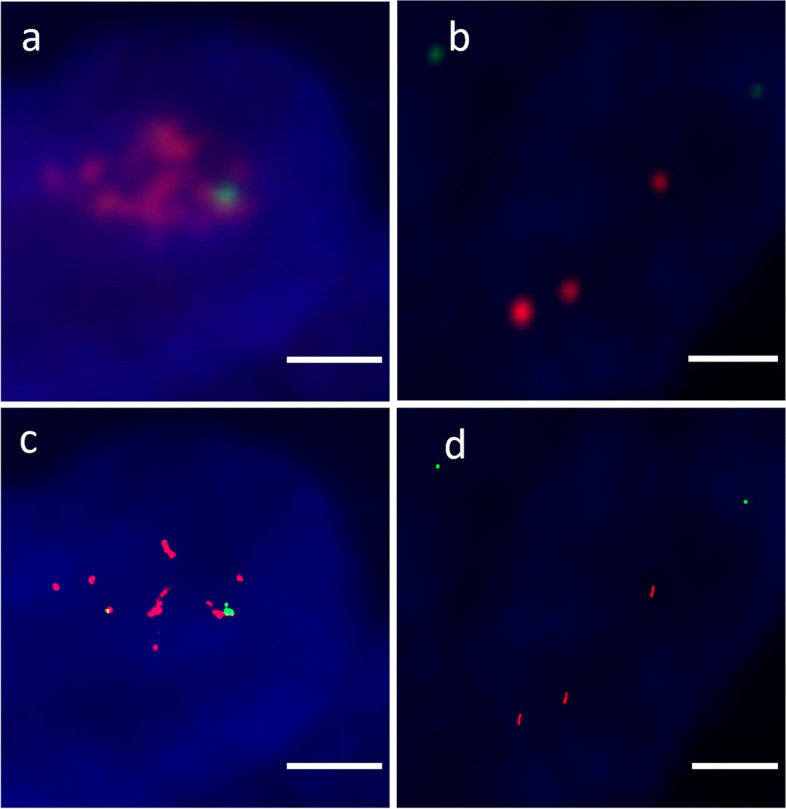
Conventional-resolution vs super-resolution images of nucleus from HER2-positive and HER2-negative tissues. *HER2* and CEP17 quantification and HER2 status evaluation in breast cancer tissue samples with dual-probe FISH for *HER2* and CEP17 amplification, where red signals indicate *HER2* and green signals indicate CEP17. Images show conventional-resolution of a single nucleus in (**a**) HER2-positive and (**b**) HER2-negative tissue, and super-resolution image of the nucleus of (**c**) HER2-positive and (**d**) HER2-negative tissue. The HER2 status was determined by routine FISH. The scale bar is 2 μm

Quantification of *HER2* and CEP17 signals conventional-resolution and super-resolution images were compared using a single nucleus from HER2-positive and HER2-negative tissues (Fig. 3). In both conventional-resolution and super-resolution images, three *HER2* signals and two CEP17 signals were observed in a single nucleus from HER2-negative tissue. In contrast, in a single nucleus of HER2-positive tissue, *HER2* signals were not quantifiable and only one CEP17 signal was visible by conventional-resolution, whereas 12 *HER2* signals and one CEP17 signal were observed using super-resolution. Table 2 describes the average *HER2* and CEP17 signal count from the 14 tissue samples. In routine *HER2*-negative samples, total counted signals of *HER2* and CEP17 had significant difference between conventional-resolution and super-resolution methods. In conventional-resolution images, there were three *HER2*-negative samples and five *HER2*-positive samples with ambiguous *HER2* signals, and five *HER2*-negative samples and two *HER2*-positive samples with ambiguous CEP17 signals. In super-resolution images, all *HER2* signals and CEP17 signals were countable in all samples.

**Table 2:** Comparison of *HER2* signal quantification using dual-probe FISH between conventional-resolution and super-resolution image techniques

	**Conventional-resolution images**^**a**^	**Super-resolution images**	***p*-value**^**b**^
Routine HER2-negative,^c^ median (min, max)			
Nuclei with HER2 signals	10 (3;27)	12 (10; 29)	0.08
Nuclei with ambiguous *HER2* signals	0 (0, 5)	0	0.15
* HER2* signals	21 (7; 50)	34 (22; 62)	0.03
* HER2* signals per nucleus	2.10 (1.85; 3.5)	2.82 (1.83; 3.93)	0.05
Nuclei with CEP17 signals	6.5 (2; 22)	10 (6; 27)	<0.01
Nuclei with ambiguous CEP17 signals	2 (0; 5)	0	0.02
CEP17 signals	10 (3; 41)	18 (11; 49)	<0.01
CEP17 signals per nucleus	1.64 (1.00; 2.00)	1.70 (1.27; 2.50)	0.37
* HER2*/CEP17 ratio	1.30 (1.02; 2.40)	1.72 (0.80; 2.36)	0.15
Routine HER2-positive,^c^ median (min, max)			
Nuclei with *HER2* signals	14 (2; 20)	15 (2; 29)	0.23
Nuclei with ambiguous *HER2* signals	12 (1; 13)	0	0.01
* HER2* signals	70 (8; 119)	134 (15; 162)	0.07
* HER2* signals per nucleus	4 (3.50; 7.44)	7.5 (4.79; 10.80)	<0.05
Nuclei with CEP17 signals	5 (2; 13)	10 (2;22)	0.53
Nuclei with ambiguous CEP17 signals	0 (0; 1)	0	0.18
CEP17 signals	8 (4; 28)	20 (4; 49)	0.54
CEP17 signals per nucleus	2.00 (1.60; 2.15)	2.00 (1.80; 3.06)	0.57
* HER2*/CEP17 ratio	2.00 (1.79; 2.13)	2.73 (2.16; 5.61)	0.23

CEP17 Chromosome 17 centromere, *FFPE* Formalin-fixed paraffin-embedded, *FISH* Fluorescence in situ hybridization, *HER2* Human epidermal growth factor receptor 2, *max* Maximum, *min* Minimum

^a^The image captured by HM-1000 fluorescence microscope without super-resolution processing

^b^Calculated by Wilcoxon signed-rank test

^c^Defined by routine FISH and IHC results following routine diagnostic criteria

The counted signals classify HER2 FISH amplification in 5 groups basing on the criteria of 2018 ASCO/CAP HER2 testing guideline update (Table 3). Three samples were classified as group 2, previously known as monosomy by super-resolution images and two of them were classified as HER2 non-amplification from conventional-resolution images.

**Table 3:** Contingency table of super-resolution and conventional resolution FISH image evaluation

		Super-resolution FISH image evaluation				
		Group 1	Group 2	Group 3	Group 4	Group 5
Conventional-resolution FISH image evaluation	Group 1	3				
	Group 2		1			
	Group 3					
	Group 4					
	Group 5		2			5
	Unevaluable	2				1

The observed *HER2* and CEP17 signals categorize samples in five groups according to the 2018 ASCO/CAP HER2 testing guideline update; Group 1 (HER2/CEP17 ratio ≥ 2.0, HER2 signals/cell ≥ 4.0), Group 2 (HER2/CEP17 ratio ≥ 2.0, HER2 signals/cell < 4.0), Group 3 (HER2/CEP17 ratio < 2.0, HER2 signals/cell ≥ 6.0), Group 4 (HER2/CEP17 ratio < 2.0, HER2 signals/cell 4.0 - 6.0), Group 5 (HER2/CEP17 ratio < 2.0, HER2 signals/cell < 4.0). Group 1 defined as ISH positive and Group 5 as ISH negative. Group 2 – 4 required further evaluation by IHC

The overall HER2 status of such cases did not change because the group 2 samples were further examined by IHC and judged negative from IHC 1+ results.

**Fig. 4:**
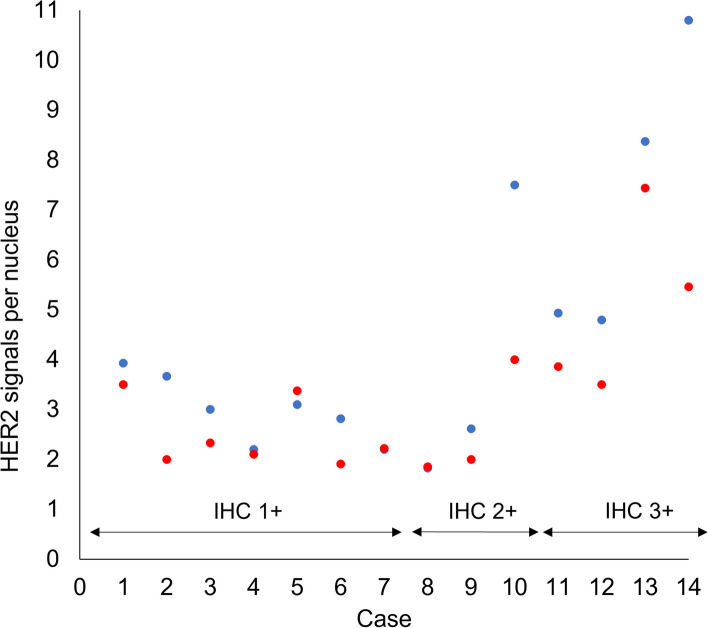
Dot plot of *HER2* FISH signals quantified by conventional-resolution and super-resolution. Conventional-resolution (red dots) and super-resolution (blue dots). The *HER2* signals per nucleus were calculated by dividing the number of *HER2* signals by the total number of nuclei counted in each image

There were three IHC 2+ samples included in this study; using routine FISH, two were diagnosed as *HER2* negative and one was diagnosed as *HER2* positive. The IHC category and *HER2* signals quantified by super-resolution images were correlated (Fig. 4).

### Patient follow-up and FISH status

**Fig. 5:**
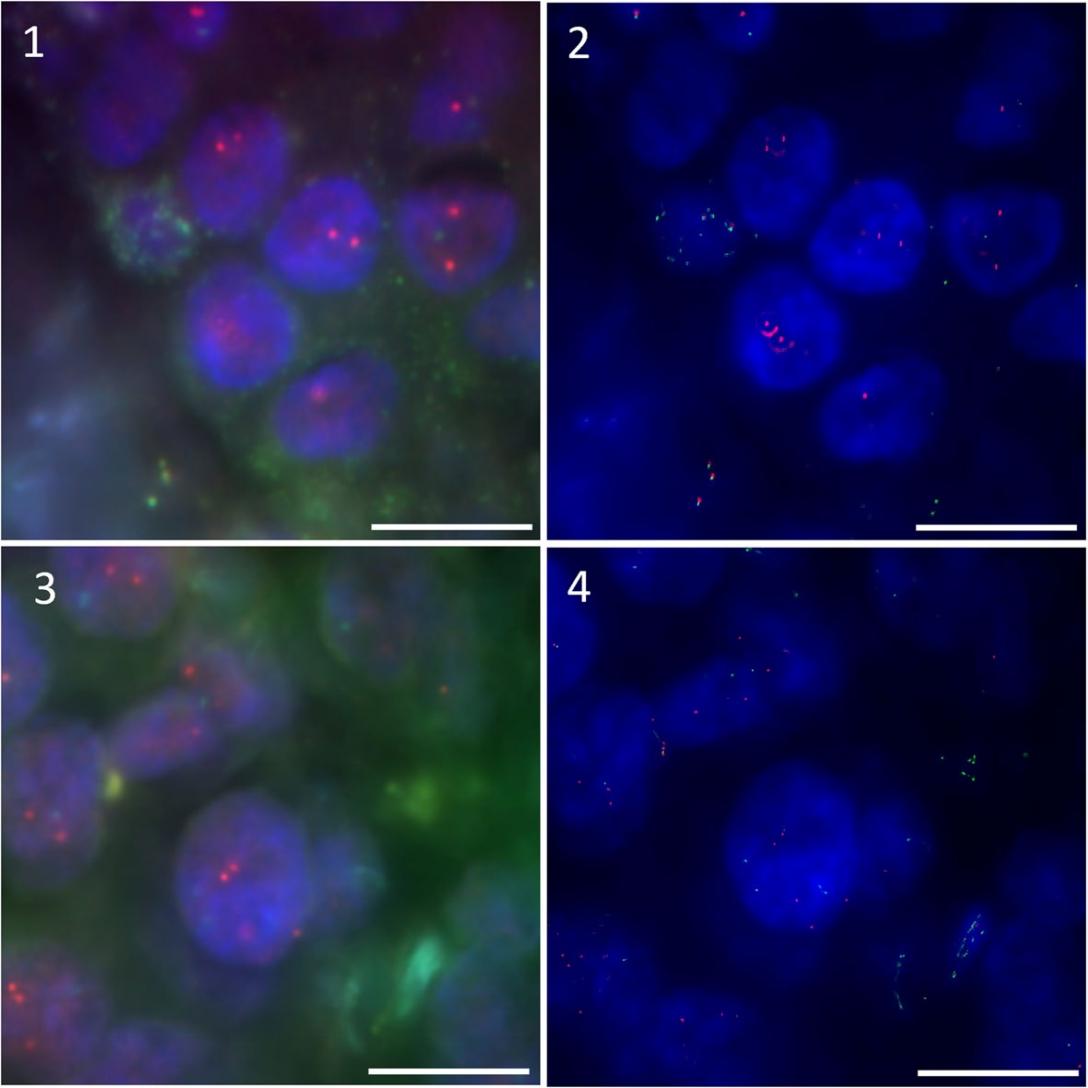
Conventional-resolution vs super-resolution images from Letrozole non-responded relapse cases. Red signals indicate *HER2* and green signals indicate CEP17. Images show the case classified as Group 2 by both resolution in (1) conventional-resolution and (2) super-resolution, and the case classified as group 2 by super-resolution image but as *HER2* non-amplification by conventional-resolution image in (3) conventional-resolution and (4) super-resolution. The signals in super-resolution are much sharper than conventional-resolution. The scale bar is 10 μm

Two of 14 patients did not respond Letrozole treatment and relapsed later. The comparison between clinical follow-up data and HER2 FISH amplification demonstrated that these two samples were classified as group 2 using super-resolution images. One of them were classified as group 2 by super-resolution image but as *HER2* non-amplification by conventional-resolution image (Fig. 5).

## Discussion

During the decade after the 2013 ASCO/CAP guideline was published [17], there was a lack of clear guidance around how to diagnose non-classical *HER2* FISH patterns. The 2018 ASCO/CAP HER2 testing guideline update provided a clear guidance of Group 1 to 5 diagnosis [1], which indicates additional IHC and FISH evaluation for non-classical *HER2* FISH patterns. On the other hand, the correct quantification of FISH signals and the solution of further improvement of FISH images were not tackled so much. In this study, we aimed to use super-resolution fluorescence microscopy for *HER2* signal quantification, with an expectation of contributing better evaluation of HER2 status.

This study showed a significant difference in *HER2* signal quantification between using conventional-resolution and super-resolution images from the HM-1000 fluorescence microscope. Super-resolution images provided better visualization of blurred and overlapped FISH signals, which are particularly common in HER2-positive samples. When evaluating CEP17 signals, some samples showed faint signals that were difficult to identify in conventional-resolution images but were countable in super-resolution images. For this reason, there was a significant difference between conventional- and super-resolution images in the number of nuclei with countable CEP17 signals. The better visualization was the result of the improvement of resolution, as well as a reduction in autofluorescence. Some samples can have high levels of autofluorescence and background noise, which can lead to confusion of the probe-specific and non-specific signals. In addition to the original function of HM1000, the Noise2Void and rolling-ball algorithms, together with machine learning, reduced the effect of autofluorescence and corrected the defocused signals in the captured images, while the ThunderSTORM plug-in acquired super-resolution images using the point-spread function of the signals from the individual molecules.

The improvement in resolution may also have a role in the accurate detection of centromeric copy number, which appears to be an important factor in chromosomal instability (CIN). In studies of breast cancer samples [18] or multiple tumor types [19], gains and losses in DNA were reported to lead to CIN and aneuploidy. The super-resolution method of HER2 quantification could be useful in detecting CIN, which seem to be associated with poor prognosis and low treatment response in patients with breast cancer [18].

We observed that in HER2-positive samples, there was a significant difference in the number of counted *HER2* signals per nucleus between conventional- and super-resolution images, but according to the current *HER2* classification, all samples with >4 *HER2* signals are considered positive, with no further classification based on the quantity of *HER2* signals above this level. However, other studies have indicated that high levels of *HER2* amplification may predict pathological complete response (pCR) to anti-HER2 therapy in patients with breast cancer. A study by Singer and colleagues showed that early breast cancer patients with a *HER2*/CEP17 ratio of >6 had significantly higher rates of pCR to neoadjuvant trastuzumab treatment than those with low *HER2* amplification levels [20]. In a similar study by Choi and colleagues, breast cancer patients with pCR to neoadjuvant trastuzumab + pertuzumab treatment had a median *HER2*/CEP17 ratio of 7.08 and a median *HER2* copy number of 17, whereas patients without pCR had a median ratio of 4.70 and a median copy number of 12 [21]. Antolín and colleagues subsequently demonstrated a significant direct correlation between pCR to neoadjuvant chemotherapy + trastuzumab and high *HER2* amplification, with pCR rates of 65% in patients with a *HER2*/CEP17 ratio of >5 and 61% in those with >10 *HER2* signals per nucleus [3].

Also, the better quantification by super-resolution images did not change the final HER2 status in this study because the samples which are not clearly positive or negative with observing FISH amplification, so-called non-classical *HER2* amplification were examined by IHC and judged by IHC result. We observed two samples which changed their *HER2* FISH classification from negative to monosomy which is non-classical *HER2* amplification by super-resolution method. In future investigation, we would like to evaluate significant number of samples to confirm such FISH classification change.

This result suggests that super-resolution method may improve the differentiation between *HER2*-low and *HER2*-negative to better categorize the patients who can benefit from new treatments that target *HER2*. This study faced two Letrozole non-responded relapse cases which coincide group 2 according to the 2018 ASCO/CAP definition. This could provide a spark for HER2-low definition to correlate the clinical outcome. There are several ongoing studies in patients with *HER2*-low breast cancer, several of which are exploring the minimum HER2 expression threshold required for drug efficacy [22]. In this regard, a more precise study of the impact of gene copy number is also warranted.

Several studies have examined whether automated quantification can improve the inter-observer variability of HER2 [23] and Ki67 [24] amplification in breast cancer samples. These studies both used machine-learning technology for quantitative digital image analysis that resulted in excellent inter-observer reproducibility and concordance with pathologist assessment [23, 24]. In future studies, we plan to evaluate whether we can apply similar technology to automatically quantify *HER2* amplification in breast cancer patients using super-resolution HM-1000 images.

This study had some technical limitations. The routine sample preparation protocol in hospital and the de-paraffination process may have caused high levels of background noise in some samples. The HM-1000 fluorescence microscope builds in four excitation lasers but does not have emission or excitation light filters, so noise-free images could not be obtained. This problem may be resolved by using a specific fluorochrome that considers the excitation wave, but commercially available *in vitro* diagnostic probes were used in this study considering to apply super-resolution method in future clinical practice with less impact for the actual routine. Machine learning and prediction techniques were utilized to reduce background noise, but these methods should be validated in future studies if the processed image only contains *HER2*- or CEP17-specific signals. Although the small sample size, we found statistically significant difference in the signal count for diagnosis. The prospective validation study with bigger rate of tumors in equivocal status could confirm the clinical application. 

## Conclusions

Overall HER2 status evaluated by conventional-resolution and by super-resolution images maintained the same; however, *HER2* FISH amplification patterns changed from negative to monosomy, and the number of counted *HER2* signals in HER2-positive samples increased with super-resolution versus conventional-resolution images. Improvements in the accuracy of *HER2* signal quantification may give clinicians further information regarding *HER2* expression status and potentially allow for improved precision with regard to therapeutic decisions especially in patients with HER2-low breast cancer.

## Data Availability

The data that support the findings of this study are available within the paper. Raw data files are not openly available due to reasons of sensitivity and confidentiality.

## Abbreviations

HER2: Human epidermal growth factor receptor 2
FISH: Fluorescence in situ hybridization
IHC: Immunohistochemistry
CEP17: Chromosome 17 centromere
ASCO: American Society of Clinical Oncology
CAP: College of American Pathologists
DAPI: 4',6-diamidino-2-phenylindole
SSC: Saline sodium citrate
SD: Standard deviation
CIN: Chromosomal instability
pCR: Pathological complete response
